# Realizing a human-centered digitalization of the energy sector

**DOI:** 10.12688/openreseurope.15129.1

**Published:** 2022-12-15

**Authors:** Heather Jean Arghandeh Paudler, Valeria Jana Schwanitz, August Wierling

**Affiliations:** 1 Department of environmental sciences, Western Norway University of Applied Sciences, Sogndal, 6856, Norway

**Keywords:** human-centered digitalization, FAIR and open data principles, digitalization, energy transition, low carbon energy transition, citizen involvement, policy support

## Abstract

The digitalization of the energy sector is in progress, but Europe is about to fall behind. A human-centered design approach ensures that it takes place with, and for the benefit of, people, for which this letter puts forward policy recommendations. People need access to tailored and ready-for-use tools that help the realization of societal co-benefits, bring individual gains, remove human burdens, and ensure just participation of all societal groups. Substantial additional intellectual and financial resources are required for the development of digital tools and products to seize opportunities for citizens to engage, in the energy transition, optimize energy consumption, and manage active participation.

## Plain language summary

The digitalization of the energy sector is in progress, but Europe is about to fall behind. It is important that the energy transition takes place with, and for the benefit of, people. We provide policy recommendations in this letter. People need tools that are easy to access, ready-to-use, and are beneficial. It is important to ensure just participation of all societal groups. To achieve this, a large amount of intellectual and financial resources are needed. This allows citizens to engage, lower energy costs, and actively participate.

## Disclaimer

The views expressed in this article are those of the authors. Publication in Open Research Europe does not imply endorsement of the European Commission.

## Framing a human-centered digitalization of the energy sector

### Distinguishing two human-centered perspectives

**Figure 1.**
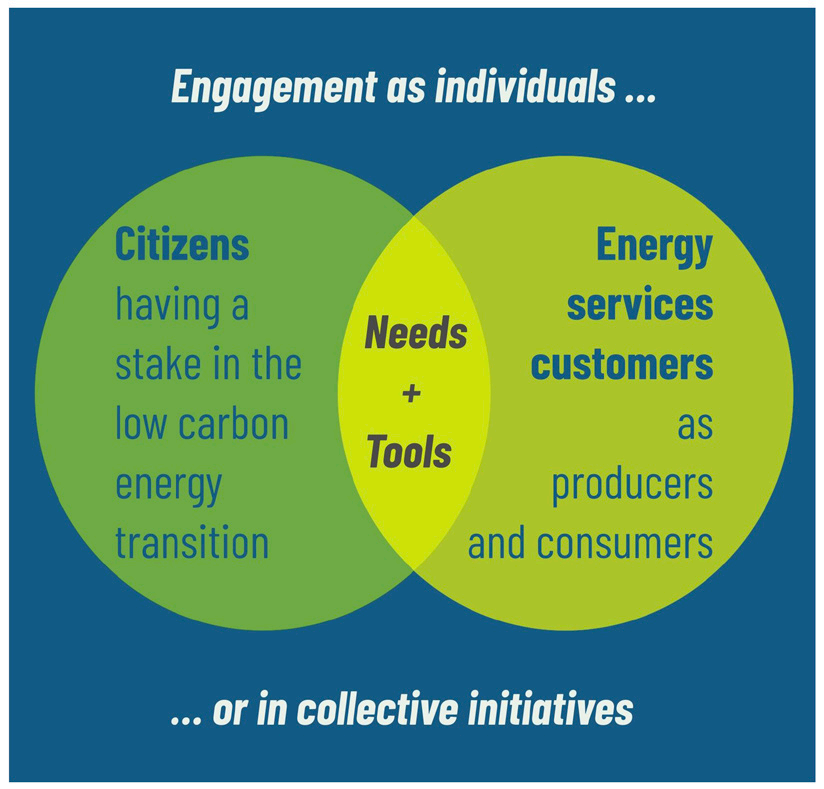
Two human-centered perspectives. Humans as customers of energy markets and humans as citizens who have a democratic stake in transition processes.

All citizens have a stake in the necessary transformation of the energy system and the digitalization of the energy sector because it affects everybody ( Figure 1). In the fundamental changes of decentralizing, decarbonizing, and restructuring the energy sector, citizens should have a say in how the transition progresses and in the way in which policies are designed and implemented. Furthermore, people are affected as customers of the energy market who pay for and demand the provision of reliable and affordable energy services which provide electricity, thermal comfort, lighting, and transportation of people and goods. Increasingly, more and more citizens also become prosumers, producing energy, selling electricity, and offering a range of energy services. This open letter proposes that the way forward in the energy transition is to design a human-centered digitalization of the energy sector.

These pioneer citizens - whether individual or collective prosumers - often engage out of curiosity. However, the majority of people only want to engage in new ways if they are convinced about the value proposition that is offered to them: they seek higher value, more utility, or better comfort (c.f. Giacomin, 2015 for an introduction to human-centered design; Shapira, Ketchie & Nehe, 2017 for connecting design thinking with strategic sustainable development; and Wilson, Hargreaves & Hauxwell-Baldwin, 2015 on review of the prospects of smart homes from a user perspective). Only with such clear offers will they actively take part in and accept revisions of the way energy services are delivered in the future. Thereby, the means to realize the offered value is of less importance; it is the mobility service itself that matters and not the possession of a car per se.

The distinction between the two human-centered perspectives has to be systematic, since their needs do not necessarily overlap. In other words, the means to engage people also differ. This is important to stress because digitalization of the energy sector only unfolds its full transformative potential if energy services are user-tailored, and citizens can truly engage in the transformation. True for both perspectives, however, is the fact that humans dislike change in general. Therefore, any approach to a human-centered transition requires answering “Why does it matter for me?”. The answers have to be provided through an evidence-based theory of change; in other words, solutions that link to a portfolio of low-entry practice changes that offer benefit. The road to engagement must accommodate for the speed and depth of the transformation as it is perceived by both perspectives.

To best realize a transition whose tools and services benefit people, the transformation of the energy system and the digitalization of the energy sector need to incorporate a human-centered design. To establish a practice of change, the involvement of both citizen-perspectives in all four human-centered design stages ( 1) is necessary. The inclusion of energy market customers and citizens only at the end of a feedback process falls short in two ways. First, the evaluation is likely without impact if the linear process is not moved into a revision cycle. Second, not involving users of tools and services from an early stage onward in a co-design process will cause inefficiencies. More time and resources will be needed to understand context, elicit requirements, develop value propositions, and design, build, and evaluate prototypes. Only by meeting people's needs will citizens benefit from the change, accept transition processes, and actively participate as prosumers and informed citizens.

### Actively engaging people

Behavioral theories have shown that needs-based knowledge about the energy system and how it functions is a precursor to the enabling of entry-points in order for citizens to actively engage and inform themselves (c.f. Huffman, 2009; Markard, 2018; Renn *et al.*, 2020). People who become prosumers in energy markets will understand to a deeper degree how the transition affects them and their lives. People who engage collectively in energy communities are able to break down the complexity of the energy transition, being equipped to propose and implement local solutions. A recently finalized inventory on collective citizen-led action in the energy transition for each country in Europe provides ample evidence for the local solutions found by over 10,000 initiatives across Europe with more than 16,000 energy projects to install renewable capacities, promote energy saving, and low carbon mobility ( Wierling *et al*., 2022). It emphasizes that creativity of solutions grows with the number of people involved.

Actively engaging people stabilizes democracy and connects people with local administration, policy institutions, and businesses. Citizen engagement depends on transparent and prompt access to reliable information and access to planning and decision-making systems ( Haf & Robison, 2020; Vieira Fernandes & Santos Silva, 2022). A human-centered digitalization is - as a first step - one that prepares the ground for people to take part in change processes. In a second step, the full-fledged digitalization of the entire energy system supports human decision and control by regulators, energy businesses, and end-users alike. With the second step, autonomous machine agents act in service of humans, supporting the monitoring and steering of dataflows and data-driven appliances for managing the energy system infrastructure. 

## Designing tools that meet peoples' needs

People need access to tailored, user-friendly, and ready-for-use tools that come with a low entry barrier ( Wilson *et al.,* 2015). Needs-based tools not only help in the realization of societal co-benefits, but also bring individual gains. Knowledge tools raise awareness of an environmentally friendly energy transition and empower people in general, but tailored, they incentivize energy and resource saving behavior and thereby lower energy costs. Digital tools can bridge the gap between professionals and laypersons, allowing average citizens to engage and even compete with incumbents of the energy market.

Two broad categories of digital tools include 1) tools to seize opportunities and prospective benefits for citizens to engage, and 2) tools to optimize (private or collective) energy consumption ( Klass & Wilson, 2016) and manage active participation in the energy markets. Tools can be further separated by technical, legal, and informational grounds. Examples for technical circumstance tools are localized solar cataster and wind power maps. They help to choose the most beneficial technology option to install in a home or community. Legal requirement tools inform on available support schemes such as retrofitting opportunities. They also provide information on how collective engagement is possible, for example how the EU directives related to energy communities are implemented in different places. User-specific information portals could contain tools that inform about policy-steered transition processes, enable scenario exploration by the users and allow observation of whether or not policy implementation is locally on track. Tools within the second category of optimization and management allow for an increase of competition in energy markets so that their liberalization benefits the people. Such tools could permit switching providers to lower cost or the ability to choose preferred fuels and also include tools to plan, monitor, and control local energy grids established by energy communities. These tools are only attainable with the right policies in place that encourage participation, set the framework, and promote businesses to develop the necessary tools.

## Setting up machines to serve people

Digitalization of society and the energy sector in particular is an ongoing process and must be steered into the desired direction and aligned with the needs of people. Besides helping society to cope with transition challenges, digitalization allows society to harvest values that lie in growing data and interconnectivity. Digitalization also allows for the transparent display of complex transition processes, empowering people to make informed choices.

A human-centered digitalization rests on machine-actionability and interoperability between energy system actors and components. This can be achieved by ubiquitous implementation of the FAIR (findability, accessibility, interoperability, and reusability) data guiding principles ( Wilkinson *et al.*, 2016), which ensure findability, accessibility, interoperability and reusability of data and information in general. The concept of open (or shared) data adds a layer which is intrinsically linked to transparency. By complementing proprietary data with novel forms of open data, new possibilities for collaboration among different agents are created. For example, if different agents in a supply chain are linked through FAIR and shared data, then resource efficiency envisioned by the circular economy can be fostered. Or digital energy community networks can more easily transfer best practices and lessons learned.

**Figure 2.**
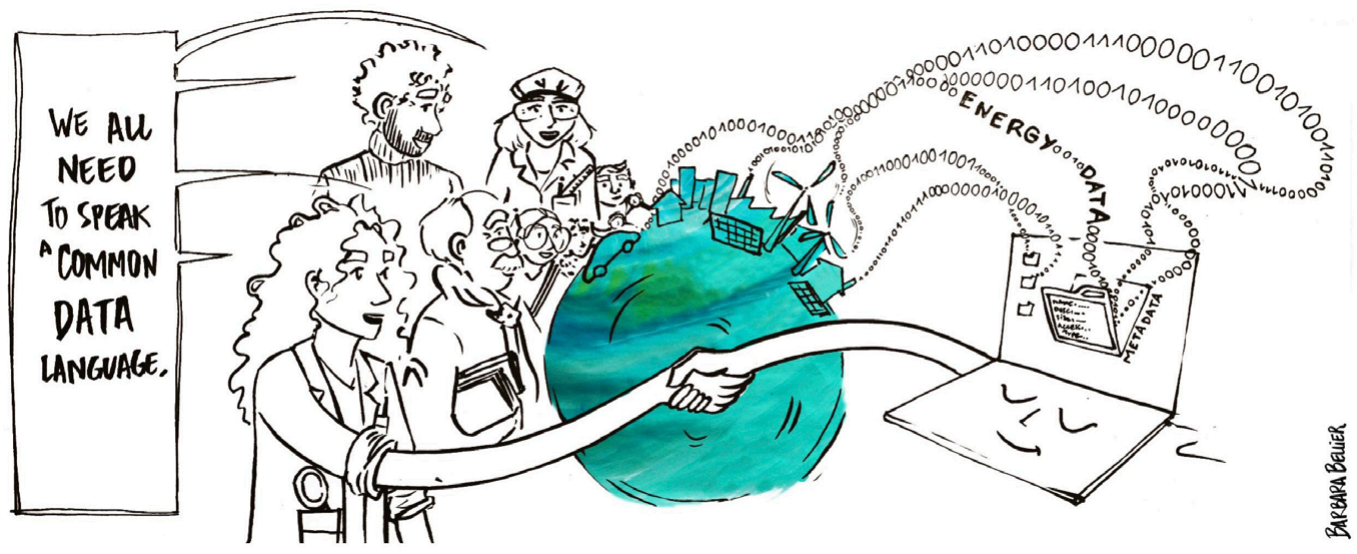
Need for human-machine communication.

An important precondition for being able to develop the necessary digital tools is a common data language that delivers seamless and automated communication between humans and machines ( Figure 2). Digitalization acts as an enabler driven by data, analytics, and connectivity between data, devices, and users. This is why all stakeholders of the digital transformation need to speak and interpret a common data language. Moreover, machines need to be in the position to understand the language humans speak (refer to Box 1 for an illustrative example of the common data language challenge). Only then can siloed data, and data with poor provenance and context information, be overcome. This would permit interoperability between all relevant data that digital tools need access to in order to function as envisioned for the low carbon energy transition.

**Box 1. Example human-machine communication**

A simple, but illustrative example is how to answer “What is 2 kg”? Humans read the question and remember that they have felt what a kilogram feels like, and they know from school what the unit means. Using the standard set by the gauging office, humans understand that 2 kg is twice as much as the normed unit. Machines, on the other end, are able to interpret statements through parsing and resolving of links between semantic fragments, bringing meaning. For all parsed elements, machines need definitions they can access from persistent websites. Today, this translation for machines still requires substantial human labor and engagement. It is by no means offering a value proposition that is attractive to engage in, neither for energy services customers nor for citizens in general, as well as researchers and businesses.

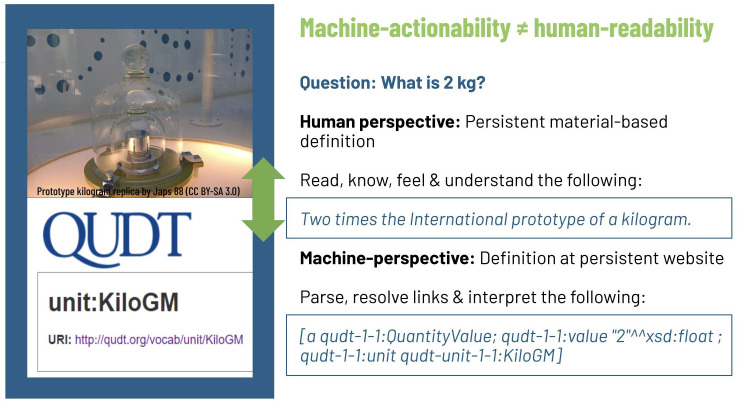

## Policies to pave the way for a human-centered digitalization

The development of human-centered digital tools, as well as the implementation of interoperability and machine-actionability of data and devices, requires substantial additional intellectual and financial resources. It is outside the scope of being accomplished in addition to the current daily tasks of policy makers, planners, businesses, academics, and most importantly, citizens. Paving the way for human-centered digitalization requires governmental support for the development of tools and products that make machine-actionability possible and that facilitate a human-centered digitalization.

Table 1 presents an overview of digital products and tools for machine-actionability, including recommendations on how policies could support the process. The focus is best placed on the development of open-source software and technology, promoted through open-source pools for energy generation and monitoring technologies that help citizens and energy communities to actively engage in the energy transition. The role of the European Commission therein can range from encouragement to prioritization, and to command-and-control measures. For example, to foster standards for open data, support markets for open technologies, enforce open metadata, and to fund experimentation with open data (shared data) based business models. Furthermore, data services and software that help to implement FAIR data guiding principles with a focus on machine-actionability should be prioritized. The key is to enable interoperability of personal, public, governmental, private sector, and research data. This can be facilitated through standard setting and the introduction of labels for FAIR metrics, leading the development of machine-actionable vocabularies, and supporting the niche markets for FAIR data services. Last, but not least, the European Commission needs to realize the tremendous effort that is needed for universal, machine-actionable implementation of the FAIR data guiding principles. This requires new educational programs, institutional capacity building, and the promotion of new job profiles, such as domain-specific data stewards.

**Table 1.** Tools and products for machine-actionability. Along with policy recommendations and call for action.

Digital products & tools	Policy role: from encouragement to prioritization to command-and-control
**Open-source software and technology**, e.g. open-source pool for energy generation and monitoring tools, Operation & Maintenance scenario explorer, customer- centric accounting software; tools supporting open energy data business models, open innovation models.	**Funds to support**: platforms that provide access, energy cooperatives and businesses that commit to open source data and tools, co-creation initiatives, shared innovation consortia; setting **standards for open data**, supporting the establishment of **markets ** **for open technologies**, enforcing **open metadata**, encourage the identification and experimentation of **open data business models** (measuring the value of data, market interaction methods, and data trade).
Data services and software that adhere to the FAIR (findability, accessibility, interoperability, and reusability) principles (i.e., implementing the **FAIR principles ** **with focus on machine-actionability**).	Supporting pioneers at different levels (e.g., services for **interoperability of personal, ** **public, government, and research data**), establishing and enhancing European AI architectures (energy data spaces, etc.), **setting standards** (FAIR data **metrics** and seals, **machine-actionable vocabulary** for metadata), **acknowledging the effort** (investing in capacity building, education, and new job profiles), supporting **niche ** **markets for FAIR data services**, funding **low-entry documentaries**, and educational material.

The list of policy recommendations in Table 2 addresses tools and products that facilitate human-centered digitalization. These include low-entry barrier tools and apps, one-stop platforms, solutions that support the functioning of energy services markets (and energy data markets), apps and tools that help in the development of new products as well as evaluation software. A central target for the European Commission should thereby be the support of energy communities and co-design principles. Energy communities have proven to be sources of social innovation (c.f., Horizon 2020 Project COMETS, 2019-2022); they successfully experiment with yet-immature technologies, and they take the role of early adopters, for example in driving wind energy development in Denmark. Moreover, they are close to the target groups for a human-centered digitalization. Support from the European Commission is needed for setting and reinforcing standards for data security and privacy, and rules for interaction in energy services and energy data markets. It starts with communicating and raising awareness of the fact that data are assets, and they should therefore be protected and stay in control of the individual. It continues with funding low-entry barrier documentation and communication of how to use the services to close the digital divide. Finally, it also includes market regulations, for example the introduction of labels for safe data standards similar to energy efficiency labeling of home devices. In summary, policy support is needed along the entire services supply chain and across stakeholder groups. Concerted effort is fundamental to steer and bring about a human-centered digitalization and to establish functioning energy services and data markets. Only then is Europe able to keep up with and compete in the global context.

**Table 2.** Tools and products that facilitate human-centered digitalization. Along with policy recommendations and call for action.

Digital products & tools	Policy role: from encouragement to prioritization to command-and-control
**Low-entry barrier tools and apps** for citizens and energy service customers (one-stop dashboard, personal data center, multifunctional tools), data hubs with machine-powered tailored analytics, open-source software for electricity trade is needed by energy communities, cost saving scenario explorer.	**Support energy communities** which accumulated relevant techno-digital knowledge and who are connected to diverse groups of citizens (e.g., encouraging outreach, education, and training activities; developing tools and apps tailored to the needs); setting and reinforcing standards for **data security and privacy**; create awareness that **data are assets** and should be protected and stay in control of the individual.
**One-stop platforms** with easy access to energy planning tools for all stakeholders (e.g., energy atlas, solar photovoltaic system video instructions).	Support platforms, best-practice forums, **energy data stewards**; fund **low entry-barrier documentation and communication** of how to use the services; prioritize citizens with low digital competences to overcome digital divide.
Tools that facilitate the **functioning of energy services ** **markets** (e.g., tools for interoperability of energy data and services, apps to determine value in data products, multi-actor communication).	Support consortia to develop these (along services supply chains, across stakeholder groups), consider **certificates/labeling for ** **market participants** (e.g., data hygienic standards, compliance with ISO 9241-210:2019); set standards for user-centric businesses and governance.
**Tools that facilitate human-centered digital services ** **development** (e.g., autonomously screening for relevant legislation and standards when developing new services; tools that allow for the evaluation of monitoring and transparency of transformation processes).	Support consortia to develop these (along services supply chains, across stakeholder groups).
**Tools for holistic human-centered evaluation** (e.g., that help to incorporate customer-centric and citizen-centric perspectives through integrated evaluation; apps, software, platforms to collect feedback that evaluate the usability of services).	Support the systematic elicitation of feedback, trade-offs, and synergies between the perspectives.

## Data Availability

No data are associated with this article

## Footnotes

1: The four principal stages are: 1) Researching & understanding context of use, 2) Eliciting user requirements and developing value propositions, 3) Designing and building of prototypes, 4) Evaluating.
