# Supplementary material for: Realizing a human-centered digitalization of the energy sector
Source: Open Res Eur. 2022 Dec 15;2:140. [Version 1] doi: 10.12688/openreseurope.15129.1 (PMC10445812; doi:10.12688/openreseurope.15129.1)
Supplement: Peer review history

# Peer review: Reviewer response for version 1

**DOI:** 10.21956/openreseurope.16367.r31861

**Published:** 2023-06-20

**Authors:** Siyu Ren

**Affiliations:** 1 Nankai University, Tianjin, Tianjin, China

1. The manuscript needs improve the quality of the introduction section, because the author should tell us why you need to analyze the problem and what is the relationship of the factors you mentioned, and which is very important to us.
2. The major defect of this study is the debate or argument is not clear stated. Hence, I would suggest the author to enhance your theoretical discussion and arrives your debate or argument.
3. I noticed that the novelty of this paper is not described in detail. The author needs to elaborate on the importance of this research topic.
4. A summary of the research gaps in the existing literature allows the reader to understand the differences in the Review.

Where applicable, are recommendations and next steps explained clearly for others to follow? (Please consider whether others in the research community would be able to implement guidelines or recommendations and/or constructively engage in the debate)

Yes

Does the article adequately reference differing views and opinions?

Yes

Are all factual statements correct, and are statements and arguments made adequately supported by citations?

Yes

Is the rationale for the Open Letter provided in sufficient detail? (Please consider whether existing challenges in the field are outlined clearly and whether the purpose of the letter is explained)

Yes

Is the Open Letter written in accessible language? (Please consider whether all subject-specific terms, concepts and abbreviations are explained)

Yes

Reviewer Expertise:

Digital economy; Environmental governance; Energy consumption; Climate policy

I confirm that I have read this submission and believe that I have an appropriate level of expertise to confirm that it is of an acceptable scientific standard, however I have significant reservations, as outlined above.

# Peer review: Reviewer response for version 1

**DOI:** 10.21956/openreseurope.16367.r31864

**Published:** 2023-06-20

**Authors:** Nicolò Rossetto

**Affiliations:** 1 European University Institute, Fiesole, Toscana, Italy

The open letter addresses an important topic, how to ensure digitalisation of energy answers the needs of people and can support societal development.

 However, the letter is quite abstract and fails to provide a paragraph setting adequately the scene, in particular from a policy point of view, and a paragraph introducing/anticipating the main elements of the reasoning/the structure of the letter itself. On top of that, the letter mentions a lot of items, including several policy recommendations, but is less clear in providing the framework supporting/justifying those items/recommendations. 

 As a result, it is not easy for a person to fully evaluate the contribution and take full advantage of it.

Where applicable, are recommendations and next steps explained clearly for others to follow? (Please consider whether others in the research community would be able to implement guidelines or recommendations and/or constructively engage in the debate)

Partly

Does the article adequately reference differing views and opinions?

Partly

Are all factual statements correct, and are statements and arguments made adequately supported by citations?

Partly

Is the rationale for the Open Letter provided in sufficient detail? (Please consider whether existing challenges in the field are outlined clearly and whether the purpose of the letter is explained)

Partly

Is the Open Letter written in accessible language? (Please consider whether all subject-specific terms, concepts and abbreviations are explained)

Partly

Reviewer Expertise:

Energy economics, energy regulation, electricity markets and grids.

I confirm that I have read this submission and believe that I have an appropriate level of expertise to confirm that it is of an acceptable scientific standard, however I have significant reservations, as outlined above.

# Peer review: Reviewer response for version 1

**DOI:** 10.21956/openreseurope.16367.r30532

**Published:** 2023-01-30

**Authors:** Rafael Mayo-Garcia

**Affiliations:** 1 Departmento de Tecnología, CIEMAT, Madrid, Spain

*The letter puts forward policy recommendations in order to properly tackle a human-centered design approach that will ensure that it takes place with, and for the benefit of, people, in Europe.*

*It clearly addresses a topic of much interest from a proper perspective and is of utmost importance if the region wants to avoid a digital gap that will leave some citizens outside of the digitalisation of the energy sector.*

*The elaboration of the arguments is quite clear and the proposed policies of interest. I recommend stakeholders take them into account.*

*The digitalization of the energy sector encompasses more topics than data curation under FAIR principles. In this sense, the letter is coherent and valid as it is, but the title should be more specific about the content reading below.*

*The letter opens the door to new contributions targeting other activities related to the digitalization of the energy sector and how it can be achieved such as simulation, AI, cybersecurity, traceability, etc.*

Where applicable, are recommendations and next steps explained clearly for others to follow? (Please consider whether others in the research community would be able to implement guidelines or recommendations and/or constructively engage in the debate)

Yes

Does the article adequately reference differing views and opinions?

Yes

Are all factual statements correct, and are statements and arguments made adequately supported by citations?

Yes

Is the rationale for the Open Letter provided in sufficient detail? (Please consider whether existing challenges in the field are outlined clearly and whether the purpose of the letter is explained)

Partly

Is the Open Letter written in accessible language? (Please consider whether all subject-specific terms, concepts and abbreviations are explained)

Yes

Reviewer Expertise:

Energy; digitalization

I confirm that I have read this submission and believe that I have an appropriate level of expertise to confirm that it is of an acceptable scientific standard.
